# The dynamic relationship between plant architecture and competition

**DOI:** 10.3389/fpls.2014.00275

**Published:** 2014-06-17

**Authors:** E. David Ford

**Affiliations:** School of Environmental and Forest Science, University of WashingtonSeattle, WA, USA

**Keywords:** stand structure, canopy structure, morphogenetic plasticity, resource acquisition

## Abstract

In this review, structural and functional changes are described in single-species, even-aged, stands undergoing competition for light. Theories of the competition process as interactions between whole plants have been advanced but have not been successful in explaining these changes and how they vary between species or growing conditions. This task now falls to researchers in plant architecture. Research in plant architecture has defined three important functions of individual plants that determine the process of canopy development and competition: (i) resource acquisition plasticity; (ii) morphogenetic plasticity; (iii) architectural variation in efficiency of interception and utilization of light. In this review, this research is synthesized into a theory for competition based on five groups of postulates about the functioning of plants in stands. Group 1: competition for light takes place at the level of component foliage and branches. Group 2: the outcome of competition is determined by the dynamic interaction between processes that exert dominance and processes that react to suppression. Group 3: species differences may affect both exertion of dominance and reaction to suppression. Group 4: individual plants may simultaneously exhibit, in different component parts, resource acquisition and morphogenetic plasticity. Group 5: mortality is a time-delayed response to suppression. Development of architectural models when combined with field investigations is identifying research needed to develop a theory of architectural influences on the competition process. These include analyses of the integration of foliage and branch components into whole-plant growth and precise definitions of environmental control of morphogenetic plasticity and its interaction with acquisition of carbon for plant growth.

## INTRODUCTION

Competition results in the preferential accrual of resources by one plant relative to its neighbors. How does plant architecture affect this process? Two characteristics of the subject determine the type of answers we can expect. First, competition and architectural development are interacting dynamic processes. As a plant grows, its architecture changes which in turn changes the surrounding environment so altering the resources available for both the plant and its neighbors–-answers should encompass architectural effects on this dynamic process and not be restricted to static descriptions of plant form. Second, competition must be assessed in the way individuals develop within stands but explanation for how architecture affects the process requires understanding of details of plant growth–-answers should encompass knowledge about both plant populations and plant growth processes and not be restricted to just one or other body of knowledge. The scope of this review is competition for light in even-aged single-species stands, such as crops and many types of naturally regenerated vegetation. Plant architecture refers to morphology and its associated physiology.

Section “The Dynamics of Stands Undergoing Competition” examines what needs to be explained about competition. The developmental sequence of single-species, even-aged stands undergoing competition is described along with metrics that can be used for this description. There has been increasing realization that variation in the competition process may be related to differences in plant architecture. Suggestions are made why the relationship between architecture and competition is not explained by competition theories based on plant population dynamics.

Section “Plants as Competitors” discusses the properties of plants as competitors. These include: resource acquisition plasticity, which enables plants to maintain dominance; morphogenetic plasticity, which can reduce the impact of being shaded; and differences in the efficiency of interception and utilization of light that can affect both the degree of dominance found in a stand and its overall productivity.

Section “Development of Theory for the Effects of Plant Architecture on Competition” outlines components for theory defining competition as the result of interactions between architectural processes and describes how it can be used, developed, and tested.

Field measurements of stands and plants undergoing competition are being combined with use of models in the analysis of architectural effects on competition. In section “Architectural Models and Competition Dynamics,” examples of such studies are discussed that improve our understanding. Future directions are discussed in section “Conclusions”.

## THE DYNAMICS OF STANDS UNDERGOING COMPETITION

Most investigations into competition in whole stands have concentrated on describing the resulting population structure. Typically, investigators have sought generalizations, often implicitly, about structures that would apply to all stands and under all conditions (e.g., White, 1981). However, although there are common features, important variations have been found between species, and conditions of stand growth, that undermine construction of general theories of competition based on population studies (e.g., Weller, 1987).

Three general features observed in stands undergoing competition are: (i) emergence of dominants and suppressed individuals that sometimes die; (ii) development of spatial evenness in large and surviving plants; and (iii) general increase in the size of surviving plants as competition-induced mortality takes place. These features have been analyzed using three empirical descriptors: plant size–frequency distributions, spatial distribution of individuals, and plant size:density relationships during the self-thinning stage.

### SIZE–FREQUENCY DISTRIBUTIONS

**FIGURE 1:**
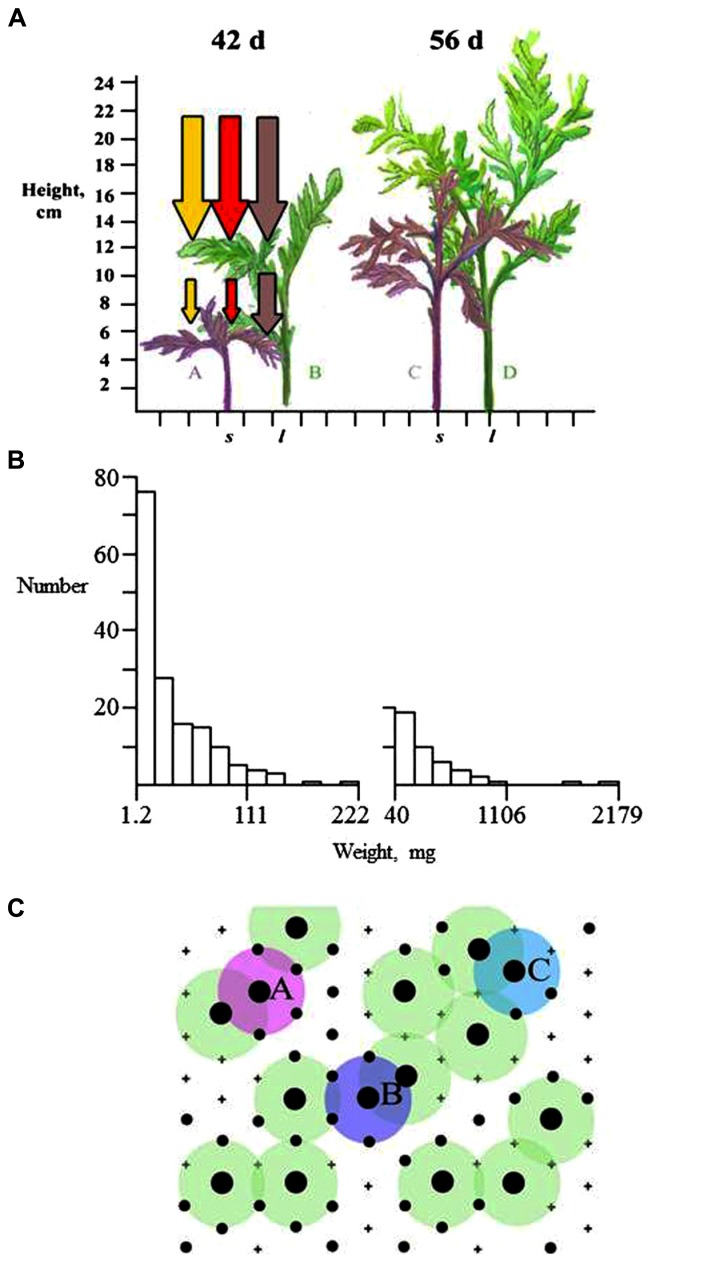
**Development of population and stand structure in *T. patula* planted in a 2 cm triangular lattice (after Turley and Ford, 2011). (A)** Plant height after 42 and 56 days showing large (C, D) and small (A, C) plants aligned on a 2 cm scale with diagrammatic reductions in PAR, and red and far-red light. New foliage grows upwards and away from the stem so that competitive interaction takes place in three dimensions. **(B)** Frequency histograms of plant dry weight at 42 and 56 days illustrating reduction in total number from 400 plants and right-skewed distributions. **(C)** The spatial arrangement of large and small plants, represented by black circles of their respective sizes at the lattice points and dead plants indicated by +. Plants **A**, **B**, and **C** are each one of a pair of large plants that are within 2 cm of each other, whereas other large plants are further than 2 cm from a large neighbor.

Size–frequency distributions of individuals within a stand are weak descriptors of competition because they do not identify the processes that contribute to stand development. As competition occurs, the frequency distribution of plant weights becomes right-skewed (**Figure 1B**), i.e., there are more smaller than large plants, originally described as log-normal by Koyama and Kira (1956). The right-skewed characteristic can be described by the Gini coefficient (Weiner and Solbrig, 1984): the differences between the weight of each of the *n* individuals, *x*, and all others are summed (numerator) and then averaged (denominator)

$$G=\frac{\overset{n}{\underset{i=1}{\sum }}\overset{n}{\underset{j=1}{\sum }}\left|x_{i}-x_{j}\right|}{2n^{2}\overset{¯}{x}}$$

*G* has a minimum of 0, when all individuals are equal, and a maximum of 1 in which all individuals but one have a value of 0.

This right-skewed distribution can be maintained through a considerable part of stand development. The inference made from this pattern, often implicit, is that large plants have greater growth and so are outcompeting smaller ones. However, the correct measurement to determine whether competition is occurring, and to assess its intensity, is the distribution of relative growth rate (RGR), weight weight^-^^1^ time^-^^1^, in relation to plant size. This provides a measure of a plant's efficiency and distinguishes from size differences that can be perpetuated in the absence of competition. In stands undergoing competition, large plants have been found to have greater RGR (e.g., Ford, 1975). The interesting feature, though little studied, is the pattern of decline in RGR with decrease in plant size (Westoby, 1982) which could provide a measure of competition intensity. Degree of skewness is also limited as an indication of competition intensity because it can be affected by mortality, a time-delayed consequence of a plant being over-topped. As small plants die, the frequency distribution of plant size may actually become less skewed (e.g., Ford, 1975; Weiner and Thomas, 1986). The relative importance of differences in RGR and mortality in producing a particular frequency distribution cannot be distinguished from trends in the Gini coefficient alone (Wiegand et al., 2008).

A further difficulty in the interpretation of frequency distributions is that they have usually been applied to measures of individual plant weight. Weight is the result of lifetime growth and so may have limited value as an indication of current status in a competition hierarchy. Nagashima (1999) suggests height may be more appropriate indicator of a plant’s status in the canopy. For *Chenopodium album* he reported that the height ranks of plants were almost fixed 1–2 weeks after canopy closure when stand height was 10–20% of its final value. Nagashima (1999) proposed three phases in community stand height development: an early phase when plants with taller or closer neighbors elongate more rapidly; a short, second phase when competition between plants affects height growth; a third phase of ~80% of stand growth when there was no change in plant height rank. This suggests a limited time in stand development when competition operates to affect establishment of a size hierarchy.

These three phases may not occur in all stands. Turley and Ford (2011) analyzed development of population structure of *Tagetes patula* using both weight and height. They showed, using a classification algorithm with the bivariate, height:weight distribution, the development of a bimodal plant size distribution with a distinct but relatively small group of dominant (large-sized) plants forming an upper canopy in the stand. These upper-canopy plants receive markedly greater illumination, and have greater RGR, than lower-canopy plants. Plants in the lower canopy do not die immediately on being over-topped and the number of plants in this mode can be three or four times that in the large-sized plant mode. In contrast to Nagashima’s (1999) analysis suggesting stability in population structure, Turley and Ford (2011) found that a further bimodal distribution develops from within the initial one. This indicates that continued development of an upper canopy is a property of a stands of this species. There are multiple references in the literature to bimodality in size–frequency distributions found in different species (reviewed in Turley and Ford, 2011) but its definition can be difficult from just one measure of plant size.

### SPATIAL STRUCTURE

In stands that have undergone competition large plants, or survivors, are spatially evenly distributed. This has been widely reported for many species and conditions of growth (Cooper, 1961; Ford, 1975; Kenkel, 1988) and indicates a process of spatial inhibition. It is the least controversial of structural properties reported for stands undergoing competition although it can take considerable time to develop to the point where it can be detected. Stoll and Bergius (2005) show the rate at which spatial inhibition develops can be affected by the intensity of competition.

Detection of spatial evenness for stands in which the initial distribution was either clumped or random, as might be expected in naturally regenerated stands or experiments using broadcast seeding, can be calculated using distance statistics (e.g., Kenkel, 1988; Bivand et al., 2013). However, the initial development of an even spatial distribution from within a clumped distribution may require more detailed analysis such as using the mark correlation function (Suzuki et al., 2008). For experiments using regularly spaced planting development of spatial evenness can be examined using lattice statistics (Bivand, 2009).

### PLANT SIZE:DENSITY RELATIONSHIP

Yoda et al. (1963) proposed a simple summary for the development of a stand during the occurrence of competition-induced mortality, i.e., the phase of self-thinning. Plant numbers per unit area, *N*, decrease due to competition-induced mortality, while surviving plants increase in mean biomass, *m*, so that

log⁡ m=γ⁢  log⁡ N+log⁡ K,

where *K* is a constant and Yoda et al. (1963) proposed that γ = –3/2. There was support for this relationship as a general result from some authors, e.g., White (1981), but through a detailed examination of available data, Weller (1987) showed there to be considerable variation. Weller suggests that a more appropriate formulation of the self-thinning relationship is

log⁡ B=β⁢ log⁡ N+log⁡ K

where *B* is the stand biomass density (g m^-^^2^), *N* is the plant density, individuals (m^-^^2^), and β and *K* are constants. β = –1/2 corresponds to γ = –3/2. Weller (1987) examined data from a large number of stands. He found some values of β not significantly different from –0.5 but some markedly so and suggested variation may be related to functional differences between species. For example, for angiosperm trees more shade-tolerant species had steeper more negative thinning slopes than intolerants, while for gymnosperm trees more shade-tolerant species had shallower slopes.

It is unfortunate that research into self-thinning largely came to a standstill following Weller’s (1987) demonstration that the self-thinning coefficient varies between species. Norberg (1988) provided an analysis of why the self-thinning slope is steeper for trees. He suggested that herbaceous plants grow with a pattern of geometric similarity, i.e., increments of branches have the same structure throughout growth. In contrast, trees have elastic similarity, i.e., branches are maintained with the same posture which requires increasing wood increment along existing branch structures, so that mean weight per plant increases more rapidly as additional space is occupied. However, this does not explain why there may be differences between shade-tolerant and -intolerant tree species suggested by Weller (1987; see also Zeide, 1985).

Important, but somewhat neglected, research by Carleton and Wannamaker (1987) suggests initial stand conditions affect the self-thinning process. In a post-fire, naturally regenerated stands of *Pinus mariana*, self-thinning occupied a distinct but limited period of stand development. While all stands went through a distinct self-thinning period, the steepness of the mortality slope was related to initial stand density and stands did not self-thin to the same final density. Stands of high initial density, ca. 194 stems per 0.01 ha, self-thinned to some 100 stems per 0.01 ha and stands with low initial density, ca. 65 stems per 0.01 ha, which is less than the final density of initial high-density stands, also showed self-thinning.

### SUMMARY DESCRIPTION OF THE COMPETITION PROCESS IN STANDS AND POSSIBLE ARCHITECTURAL EFFECTS

The most informative result from stand investigations is that large plants and/or survivors show spatial inhibition. A simple explanation is that two large plants cannot continue to grow at the same position, but in reality a complex process of spatial equalization of relative growth rates is likely required to produce this structure (Turley and Ford, 2011). Large plants are always likely to shade smaller neighbors but the crucial contest in development of spatial evenness is between neighboring large plants. The effect of competition experienced by a large-sized individual is likely to be greater when it has multiple large-sized plants as close neighbors. Plants A, B, and C (**Figure 1C**) have close contacts with another large plant and one of each pair is more likely to become suppressed than the other large plants represented in **Figure 1C**. As large plants decrease in number, the survivors would remain in a spatially even distribution. The process of resource acquisition plasticity (see section Plants as Competitors) is implicated in the asymmetric crown development that is likely to develop in these upper crowns. Spatial evenness seems to be ubiquitous where competition has occurred but differences in plant architecture could influence the rate at which crown interactions take place (Stoll and Bergius, 2005) and the depth of crown that might be involved.

**FIGURE 2:**
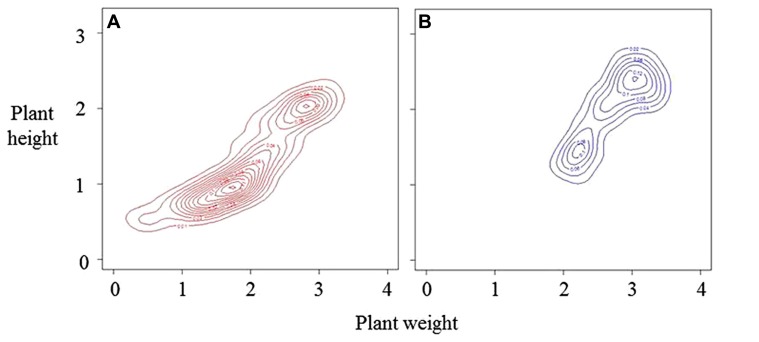
**Bivariate distributions of height and weight on the same arbitrary scales for a developing population over two time periods, A the younger stage, and **B **(based on Turley and Ford, 2011).** The distributions are represented by density estimations using kernel smoothing (Wand and Jones, 1995). Both distributions show distinct bimodality. At stage **A**, small-sized plants are the major mode. During the period of growth between **A** and **B**, some 60% of total plants died reducing the number of plants in the small-sized mode.

The process outlined in **Figure 1** results in larger plants having higher RGR and can cause formation of a distinct upper canopy and a bimodal frequency distribution of plant weights and heights (**Figure 2**). Detection of these features requires precision in measurement and analysis, and a population of sufficient size to avoid type II statistical errors. Such analyses could be used to define the effects of architecture on stand structure and productivity. For example, Vega and Sadras (2003) explicitly suggest that high productivity is associated with lack of bimodality in size–frequency distributions. Modal analysis can be made using the methods of Fraley et al. (2012).

### SOME POPULATION THEORY ON COMPETITION

Attempts have been made to classify the type of competitive interactions occurring between individuals within a stand. These have their origin in description of interactions between individuals represented as overlapping circles. Gates (1978) first suggested that one-sided competition, where the larger of two overlapping plants obtains all resources in the area of overlap, is sufficient to explain the development of bimodal populations. This approach was continued by Gates et al. (1979) who calculated how differences in crown structure may affect the area of overlap, and division of resources in the overlap, between neighboring plants. Under conditions where smaller plants may have some effect on larger ones, but a lesser effect, then competition has been termed asymmetric (Weiner and Thomas, 1986; Weiner, 1990; Schwinning and Weiner, 1998). The principal objections to these approaches are that competition is a three-, rather than two-dimensional process (Reynolds and Ford, 2005) and that within a stand there may be different types of interactions. These objections would severely limit the effectiveness in using such models in analysis of architectural effects on competition.

## PLANTS AS COMPETITORS

Three properties of plants may influence competition above ground as a 3-D process. (i) Resource acquisition plasticity, by which plants preferentially extend branches and/or foliage into regions where there are resources. (ii) Morphogenetic plasticity, by which plants respond to competition by changing their morphology, e.g., the increase in relative height growth of shaded plants. (iii) Architectural variation in interception and utilization of light: absorption of light by one plant obviously makes it unavailable for another and so will affect competition but efficiency of utilization of light in growth may also affect competition.

### RESOURCE ACQUISITION PLASTICITY

Umeki (1995) showed preferential growth of crowns of *Xanthium canadense* in the direction of resources over a growing season (0.5 plants m^-^^2^, mean plant final height 1.94 m, no mortality). Crown centers became significantly displaced from stem centers and as the population grew the spatial pattern of crown centers became regular. An index for neighborhood interference accounted for significant variation in plant RGR when calculated using crown center location, but not when calculated using stem location. Umeki (1997) developed a neighborhood model, i.e., representing plants in two dimensions, to calculate neighbor–neighbor effects. Crowns were represented as circular but with their centers moved relative to plant position to represent the asymmetry with the effect being calculated using a vector. The vector is based on the target plant’s height in relation to the height, distance and direction of neighbors. Development of crown asymmetry produced larger survivorship, larger mean size, a more regular spatial pattern of survivors and less skewness in size distributions.

Crown asymmetry has also been found in tree species. Rouvinen and Kuuluvainen (1997) found two thirds of trees in a 150–200 y *Pinus sylvestris* forest in eastern Finland had asymmetric crowns. This asymmetry was positive toward the major direction of incoming radiation, but modified by competitive status so that trees with markedly asymmetric crowns were those with free growing space and close competitors in other directions. For *Acer saccharum* Brisson (2001) found neighbors to have a strong influence on orientation of crown asymmetry. The correlation between crown asymmetry and neighbors was greatest when only size and distance of the strongest neighbor was considered suggesting that this neighbor may have a disproportionate effect on crown symmetry of the target tree.

Koike (1989) suggests that shoots develop toward brighter light without there being a phototropic effect—the growth is due to utilization of the greater available resources. For crowns of two evergreen *Quercus* species, *Quercus acuta* and the more shade-tolerant *Q. gliva*, shoot production increased with increasing light received for both species. However, the critical level of light necessary for shoot production was that a shoot should receive ~10% that of an open sky for *Q. gliva* and ~30% for *Q. acuta,* but both the numbers of shoots produced and shoot length at higher light intensities was much greater for *Q. acuta*. For both species, shoot direction was significantly affected by geotropism. The finding that branches and foliage in the upper canopy respond to greater available resources is coherent with the finding that competition produces a spatially even distribution of large plants and survivors. It also suggests that branches do not provide resources to other parts of the plant that would restrict their own growth. This can be partially explained through the concept of branch autonomy (Sprugel et al., 1991).

Branch autonomy, with respect to carbon economy, implies that branches do not import carbon they use in growth but fix it locally on the branch. Consequently, sunlit branches that fix more carbon should grow more. In their review, Sprugel et al. (1991) note three general results: (i) old shaded branches do not import carbon—maintenance respiration alone is not a sufficiently strong sink to draw carbohydrates into a branch; (ii) the internal balance of sources and sinks is such that branches are self-supporting during growing seasons; (iii) branches are least autonomous when carbon reserves are involved—particularly when substantial reserves are stored in the main stem. Local supply of carbon on a branch through current photosynthesis can be sufficient to support periods of high growth, e.g., in shoot extension, so that storage carbohydrate may not be involved. Sprugel et al. (1991) note that bud formation is a crucial process in species with determinate growth since buds, once they start to grow, can draw carbon but that the formation of buds is unlikely to be limited by carbon availability since only small amounts are required.

Branch autonomy is a useful model for considering carbon economy of established branches within an existing crown but fails as a model for describing differences in branches between trees of different sizes within a canopy (Sprugel, 2002). Stoll and Schmid (1998) found that the growth of shaded branches was less in trees that were partially shaded than in trees that were completely shaded. Sprugel (2002) offers possible explanations for this phenomenon, one being that shaded foliage on dominant trees becomes water limited when sunlit branches elsewhere on the tree are strong sinks for water. Sprugel (2002) references evidence that vascular constrictions at the base of branches keep the water system of a branch somewhat isolated from the rest of the tree so that water flow will be directed to more illuminated branches. Boonman et al. (2007) demonstrated for foliage canopies of *Nicotiana tabacum* that cytokinin in the transpiration stream affects photosynthetic rate of foliage and when transpiration is reduced photosynthesis rate declines. If this result holds for trees, it may help to explain differences in branch growth on sunlit and shaded crowns.

Resource acquisition plasticity can have two consequences for competition dynamics. In the upper canopy, it may lead to preferential expansion of foliage into places where light is not utilized and so may be the driver for continuing development of dominant plants. The extent to which this may be constrained by plant architecture has not been studied in the context of competition dynamics. A second consequence may be in reduced growth, or even death, of foliage in the lower crowns of dominant plants, but at similar height the foliage of suppressed plants is not reduced. This may aid survival of suppressed plants.

### MORPHOGENETIC PLASTICITY

Morphogenetic plasticity is the most studied plant architectural feature that may affect competition—largely due to wide interest in phytohormones. Selective absorption of red wavelengths (660 nm) by foliage relative to far-red wavelengths (735 nm; Smith, 1982; Franklin, 2008) results in decrease of the ratio of red to far red (R:FR) with increasing depth in a foliage canopy (e.g., Evers et al., 2007; Dauzat et al., 2008; Kahlen et al., 2008; Kahlen and Stützel, 2011a,b). This decrease can produce a number of morphological responses (e.g., Smith and Whitelam, 1997; Kahlen and Stützel, 2011a) mediated by the phytochrome photoreceptor system and frequently grouped together as the “shade avoidance” response (Franklin, 2008).

Morphogenetic plasticity is, primarily, a process of reaction to neighbors and may slow, or perhaps even halt, the progression toward suppression and death. Height growth is one of the most studied examples whereby plants increase height growth in response to decrease in R:FR in herbaceous plants (Ballaré et al., 1987, 1994; Ballaré and Scopel, 1997) but reduced tiller production in grasses also occurs at low R:FR (e.g., Evers et al., 2006). For *Impatiens capensis* Donohue and Schmitt (1999) suggest that primary responsive characters to changes in R:FR are increased internode elongation, decreased branch, flower, and node production, and increased meristem dormancy.

However, although effects of changes in R:FR can be demonstrated experimentally their actual effects on competition may be restricted to limited conditions. Casal et al. (1986) showed a photosynthetically active radiation (PAR) requirement response to reduced R:FR decrease. They grew *Paspalum dilatatum* and *Lolium multiflorum*, grasses of the Argentinian pampas, at different densities (2.0–31.9 plants m^-^^2^ for *P. dilatatum*, 39.8–116.3 for *L. multiflorum*) and illuminated some individuals with red light at the plant base which stimulated tillering at low but not high densities. Casal et al. (1986) indicate that limits for tillering are established by: (i) insufficient PAR in very dense canopies, which may result in reduced resources for growth; (ii) insufficiently low R:FR in sparse canopies (see also Monaco and Briske, 2001). An exception to the lack of an R:FR effect at low plant densities may be where FR is reflected horizontally so that R:FR is reduced by an increase in FR by reflection from neighbors rather than by a decrease in R through absorption of vertical beams (Ballaré et al., 1988). This effect may enable anticipation of canopy competition.

There is dispute whether height growth stimulation produced by decrease in R:FR has a cost in terms of reduced biomass growth in other parts of the plant. Maliakal et al. (1999) working with *I. capensis* and Ballaré et al. (1991) with *Amaranthus quitensis* both report that stimulation of height growth does not cause a reduction in root or leaf growth. On the other hand, Vermeulen et al. (2008a) found through experimental manipulation of R:FR, an increase in petiole length and petiole mass for *Potentilla reptans* but decrease in root, stolon, and total biomass. They also report that petiole length can be limited by the productive capacity of plants. Direct application of gibberellic acid to stems of *Phaseolus vulgaris* produced the expected increase in stem elongation but a concomitant reduction in total mass, pod number, and pod mass (Cipollini and Schultz, 1999).

Whether there is a carbon cost to R:FR-induced height growth could be important in considering its possible effect on competition. Dudley and Schmitt (1996) manipulated R:FR supplied to seedlings of *I. capensis* to produce stem-elongated and non-elongated plants. These seedlings were transplanted to within both high- (plants 3 cm apart) and low- (20 cm apart) density arrays. Lifetime fitness was calculated as number of reproductive structures produced over the lifetime of the plant. Elongated plants were more fit at high density and non-elongated plants at low density. Dudley and Schmitt (1996) suggested the advantage of elongated plants at high density was that greater height resulted in greater light capture, while their disadvantage at low density may be the result of the additional carbon cost of increased height. In a comparison of eight genotypes of *P. reptans* grown in a mixture to form single-species stands, Vermeulen et al. (2008b) found that genotypes with relatively more leaves in the top layer of the canopy were, on average, more efficient in light capture per unit leaf weight.

Photomorphogenetic-induced height increase can change plant population structures through increasing survival of smaller plants. However, the extent of this effect on the competition process, as a whole, is likely affected by the conditions of growth, particularly spacing, and the genetic structure of the population. We need to know the balance between change in R:FR in relation to PAR level for particular instances and to define how this relationship may change as the plant stand increases in height and total foliage amount. Interestingly, Dudley and Schmitt (1995) showed that while populations of *I. capensis* from more open conditions, where there was likely considerable competition for light, showed photomorphogenetic-induced height growth, populations from more shaded conditions did not. Schmitt (1997) cites additional examples of similar ecotypic variation. Certainly, we can anticipate limitation to the effect that a decrease in R:FR may have in rescuing the growth of over-topped plants since we know that small plants do die due to the effects of competition.

### ARCHITECTURAL VARIATION IN INTERCEPTION AND UTILIZATION OF LIGHT

Much research has been conducted into variation in plant structure on light absorptance but primarily with a standpoint of examining the efficiency of whole canopies rather than possible effects on competition within a stand (Niinemets, 2010). However, there are reasonable grounds for considering that architectural variation found between competing plants may influence competition through their effects on interception and utilization of light, e.g., Bendix et al. (2010). Leaf angle and the spatial distribution of leaf biomass can be affected by plant density (Vandenbussche et al., 2005; van Zanten et al., 2010) as well as leaf orientation, e.g., in maize (Drouet and Moulia, 1997) and cucumber (Kahlen et al., 2008). Some rosette species show hyponastic leaf growth in response to crowding, i.e., where leaves bend upwards, which can reduce the impact of competition: Pierik et al. (2003) planted hyponasty loss-of-function transgenic plants along with wild-type *Nicotiana tabacum* at the rosette stage of development and demonstrated that the transgenic was outcompeted.

Anten and Hirose (1998) calculated light absorption by individual plants in natural monospecific stands of *X. canadense*, a fast-growing, shade-intolerant annual. Dominant plants absorbed more light both per unit leaf area (Φ_area_) and per unit mass (Φ_mass_) and that the greater Φ_area_ more than compensated for the lower leaf area ratio of dominant plants. They concluded that the greater Φ_mass_ of dominant plants is quantitative evidence that success in competing for light is disproportionally related to the size of shoots. The proportion of mass in leaf lamina, the leaf mass ratio (LMR), decreased with increasing height but solitary plants had higher LMR than competing plants of the same height. Anten and Hirose (1998) concluded that LMR is not determined by biomechanical constraints but results from a plastic shift in allocation in response to competition.

From the perspective of competition, the production of foliage has two functions: certainly one is production of photosynthate for growth but the other can be simply shading neighbors even if there is no net gain in photosynthesis to the producing plant. Trends in development of *Zea mays* hybrids provide an example where reduced competition may contribute to an increased in total crop yield. Commercial hybrids have been selected, and commercially planted, in the central corn belt of the United States (Duvick et al., 2004) at increasingly closer spacing from ~30,000 plants ha^-^^1^ in the 1930s to ~75,000 plants ha^-^^1^ or higher by the 1990s. Production has increased markedly over this period and while a number of phenotypic characters have changed (Duvick et al., 2004) whole canopy efficiency (yield per ground area) has increased. Ford et al. (2008) compared the major hybrid used in the 1960s with that in the 1990s and found the 1990 hybrid had smaller leaves. Maize leaves are curved and Ford et al. (2008) found less curvature and more uprightness for the 1990 hybrid compared with that for the 1960 hybrid and so, potentially a reduced competitive influence. Interestingly, experimental manipulation of foliage of the same 1960s hybrid, by tying leaves into a more upright position, produced an increase in yield (Pendleton et al., 1968). Curvature is a plastic character in response to plant density and while Ford et al. (2008) found both hybrids had less curvature at higher planting density the effect was greatest for the 1990 hybrid. Fellner et al. (2003, 2006) show that this plasticity is related to an interaction between light quality and auxin particularly in the development of the leaf auricle.

How can a decrease in leaf size and an increase in leaf inclination increase productivity of a whole stand and do these changes reduce competition between individuals? Generally, increased leaf inclination from the horizontal will reduce incident quantum flux density on the leaf surface although the exact effect depends upon sun angle, foliage inclination, and the azimuth between sun and plane of the laminar surface. The effect of more upright foliage may be more appropriately thought of as reducing the duration of high incident quantum flux density.

Lateral extension of uppermost leaves may reduce light received by neighbors, but that light may be intercepted by the larger plant at intensities in excess of the light saturation point of the photosynthesis curve. An increase in inclination of these leaves, and reduction in their size, may reduce total light intercepted but not necessarily the total amount of whole-plant photosynthesis since uppermost leaves may still receive light close to the light saturation point of photosynthesis for considerable periods and more light will reach lower, more shaded, foliage. Reduced exposure of foliage to high irradiance may reduce the possibility of photo-damage and/or high evaporative demand on the foliage, e.g., Falster and Westoby (2003) for sclerophyllous plants.

Conifers also have consistent modifications to foliage in response to differences in ambient light levels. In the *Pinacea* foliage, needles are clumped around the supporting shoot at higher light levels and more spread out at lower levels and this is measured by the silhouette to area ratio (STAR), e.g., Stenberg et al. (2001) for *P. sylvestris*. In the *Cupressaceae*, which has foliage in fronds, there are multiple variations in foliage and branch structure as light varies, e.g., Edelstein and Ford (2003) for *Thuja plicata*.

In summary, although investigations of plants as competitors have shown the importance of considering competition as a 3-D and not 2-D process we still lack investigations of canopy formation, particularly of the effects of differences in resource acquisition plasticity. The three features of plants as competitors (resource acquisition plasticity, morphogenetic plasticity, architectural variation in interception and utilization of light) should not be considered separately although the history of the subject shows that they have been—which is not surprising given the details of the research required.

## DEVELOPMENT OF THEORY FOR THE EFFECTS OF PLANT ARCHITECTURE ON COMPETITION

Competition theory based on studies of populations and stands has been challenged and found wanting. This is not surprising. Competition can affect the component parts of plants differently (sec Plants as Competitors) and the effect on the individual, as a whole, results from the integration of many such interactions. The size of an individual is only an approximate indication of competitive status and can neither be used to indicate the component interactions that will occur nor how their effects may be integrated.

Nevertheless, studies of stands undergoing competition provide essential descriptions of what needs to be explained. They show that the process has multiple effects on stand structure in the numbers of plants that survive or reproduce, the distribution of plant sizes, and the spatial distribution of individuals. These effects are interrelated but the relationships may vary between species and conditions of growth. Unfortunately, some research into the properties of plants as competitors has selected just one component of stand development as an indicator of competition. This can lead to a biased view of the process as a whole and inadequate understanding of the role of particular properties.

This section outlines a theory for analyzing the dynamics of architectural effects on competition. However, prior to that it is necessary to define what the theory should be able to explain both in general terms and in details of stand development.

### WHAT SHOULD THE THEORY BE ABLE TO EXPLAIN?

There are considerable challenges in establishing a relationship between plant architecture and competition. Plant morphology is diverse and this presents us with a conundrum. If competition for light is ubiquitous then why has there not been evolution for an obviously successful architecture? Niklas (1997) suggests the requirement to conserve water reduced phenotypic options available to the earliest land plants. However, once this adaptive hurdle was overcome the next requirement was to achieve effective performance of multiple functions simultaneously—such as maximizing both light interception and reproductive success and ensuring mechanical stability—which took place where plants were growing together in communities. This increased the number of phenotypic options that had equivalent relative fitness. A system with a single defined task has fewer alternative designs compared to that for systems with manifold tasks which may be globally efficient yet comparatively poor at doing any one task. This implies there are multiple answers to the apparently simple question of what makes an effective competitor and a theory for the effects of plant architecture on competition should be required to show how different combinations of features may have similar results.

Population- and stand-level studies illustrate that the intensity of competition and the results it produces change over time. For practical purposes, this requires that investigations and studies should be assessed on rates of change rather than outcomes at a single point in time. Consider a study that compares plants with different architectures, e.g., the pioneering study by Geber (1989) which compared effects of differences in morphology between two species of *Polygonum* on competition. We can ask:

1. Is there a difference in the rate with which spatial inhibition develops? An advantage of this test is that spatial inhibition is the most reliable indication that competition has occurred. Disadvantages are that spatial inhibition may take considerable stand development before it is apparent and that use of distance statistics requires care (Loosmore and Ford,2006).
2. Are there differences in the relationship between plant size and RGR? An advantage of this test is that it is likely to indicate competition at an early stage in stand development. Disadvantages are that, generally, calculation of RGR requires repeated measurements of plants which can be difficult in stands of many plants and that we have little background information about the distribution of RGR of different components of plants, e.g., height, weight, foliage area.
3. Are there differences in rates of change in the frequency distributions of plant sizes? Some difficulties of using frequency distributions have already been discussed. Use of the bivariate plant height: plant weight distribution has the advantage of providing more information about stand structure than univariate distributions.

In practice, competition has multiple effects and more than one metric should be used. Techniques for multi-criteria assessment are discussed by Reynolds and Ford (1999) specifically for competition; Ford and Kennedy (2011) for FSPMs; Kennedy and Ford (2012) as a general strategy of investigation.

### STRUCTURE FOR A THEORY TO INVESTIGATE THE EFFECTS OF PLANT ARCHITECTURE ON COMPETITION

Five groups of postulates are required to analyze the effects of differences in architecture on competition. These are given in general terms here and specific postulates would need to be developed for particular questions.

#### Group 1

Competition for light takes place at the level of foliage and foliated axes rather than whole plants—save for small plants that have only a single foliated axis.

This is the foundation postulate for attempts to explain competition through architecture and provides a clear distinction from population or whole-plant-based theories. It ensures that explanations for differences that we may see in competition will be sought in differences in the processes of plant growth. A corollary is that the effects on the whole plant depend upon integration of affects across all foliage and foliated shoots (see Group 4). Section “Architectural Models and Competition Dynamics,” on modeling illustrates the importance, and difficulty, of explaining how this integration in the growth of the whole plant takes place.

#### Group 2

The outcome of competition is determined by interaction between two processes:

• *Exertion of dominance* through growth of foliage or a foliated axis that intercepts light that would otherwise could be utilized by neighboring foliage and;
• *Reaction to shading* through changing form and/or physiological characteristics.

This postulate is fundamental to determination of the dynamics of the competition process. Exertion of dominance can occur through resource acquisition plasticity and may be affected by efficiency of interception and utilization of received PAR. Reaction to shading can be through morphogenetic plasticity. Both process *Exertion of dominance* and *Reaction to shading* may occur on different parts of the same plant (see Group 4) and the results for plant growth and/or survival depend upon the integration of effects. It is important that studies claiming to define competition as some result of a particular architecture should define both processes. We have many studies (see Plants as Competitors) demonstrating that one or the other of these processes occur but their effects on the competition process require analysis of both components in the dynamic system.

The implication of specifying competition in this way is that the primary process is the exertion of dominance and that morphogenetic plasticity is a *reaction* to that but does not halt it or stop its effects completely. So we can expect to see stand structural characteristics that indicate competition has occurred even in roseate plants such as *Arabidopsis* (e.g., Stoll and Bergius, 2005). The intensity of competition might be considered as the extent to which *Exertion of dominance* exceeds *Reaction to shading*. The rate at which these two processes proceed may change during stand development. This is the central group of postulates that defines the work to be done to develop understanding of architectural effects on competition because it indicates the dynamics of interaction.

#### Group 3

Architectural properties of a species determine both *Exertion of dominance* and *Reaction to shading.*

Comparative analysis of species seems to be an important approach to analyze the effects of architecture on competition. Research has shown (see Plants as Competitors) that plant species exhibit different responses to being crowded but analysis of architectural effects on competition requires that exertion of dominance and responses to shade be quantified simultaneously.

#### Group 4

For an individual plant, the outcome of competition depends upon integration of effects of *Exertion of dominance* and *Reaction to shading* across the component foliage and foliated shoots.

Individual plants may simultaneously exhibit, in different parts, both resource acquisition plasticity, differences in interception and utilization of PAR, and forms of morphogenetic plasticity depending on their size, modularity of construction and architecture. In Section “Architectural Models and Competition Dynamics,” the importance of understanding the integration of plant growth is illustrated.

#### Group 5

Mortality is a time-delayed response to suppression. It is an important result of competition, particularly in dense stands or those where competition persists for long periods as in stands of trees. However, it is not generally studied in relation to architectural effects.

## ARCHITECTURAL MODELS AND COMPETITION DYNAMICS

Simulating competition provides an excellent test for functional–structural plant models (FSPMs; Godin and Sinoquet, 2005) because it requires effective representation of plants as conditions change. Two types of problems have been encountered: how to represent the integration of plant function and how to define precise relationships for operation of morphogenetic plasticity.

### INTEGRATION OF WHOLE-PLANT FUNCTION

Trees are interesting subjects for the study of plant competition. Their size and longevity raise questions about how effects of different parts of trees and how the effect of such competition is integrated in the growth of the whole tree.

Sorrensen-Cothern et al. (1993) investigated the extent to which morphogenetic plasticity affected competition. They simulated competition for light in a young stand of dense, naturally regenerated *Abies amabilis.* The model simulated growth of each individual tree in annual height and branch, including foliage increments, and the 3-D spatial location of branch and foliage was calculated.

Light was considered in contiguous vertical columns and absorption depended upon the total leaf area density and its interception characteristics that had morphogenetic plasticity depending on whether the tree was classified as a “sun,” “intermediate,” or “shade” individual, according to its relative height in the stand. No direct calculation of photosynthesis was made but a conversion efficiency, which varied between the three trees classes, was applied to the light absorbed to give a surrogate variable for photosynthate. The accounting system for the penetration and absorption of light, represented in vertical columns, allowed for spatial variability of light to have an effect on growth.

Height and individual branch increments were estimated through parameters applied respectively to the sum of the surrogate variable for the whole tree and the branch being considered. Branches grew as expanding fans of foliage with foliage density depending on the light level at each point within the branch, i.e., for the relevant 10 cm × 10 cm × 10 cm section. Model parameters were calculated using an extended sensitivity analysis.

When no plasticity was incorporated into the model, suppressed trees lost the structure found in empirical investigations. It was essential to incorporate plasticity in the amount of light absorbed per unit foliage as well as foliage survival in relation to light level to simulate observed changes in crown apex angles of suppressed trees, and correct crown lengths and to simulate the frequency distribution of tree heights and mortality.

In a subsequent uncertainty assessment of the model, Reynolds and Ford (1999) found it important to assign the plastic characteristics of foliage based on the local light level rather than based on classification of complete trees as sun, intermediate, or shade. The effectiveness of this model depended upon the interaction between resource acquisition plasticity and morphogenetic plasticity. Resource acquisition plasticity occurred because of the modular construction so that branches extended and grew into areas of greater illumination. This occurred through the depth of the canopy.

To simulate tree mass, further architectural information is required, particularly details on the structure of the tree body, i.e., the development of branches by increasing order, and representation of how branches thicken. Sievänen et al. (2008) developed LIGNUM (Perttunen et al., 1996) parameterized for *P. sylvestris* based on growth of successive metamers (Room et al., 1994) each comprising a woody pipe in the modular segment (node plus internode) terminated with apical and axillary meristems and covered with needles. LIGNUM was designed and. Wood increment to the body of the tree was calculated using the pipe-model theory (Shinozaki et al., 1964) which specified that the amount of foliage carried on a shoot section was matched by the cross-sectional area of sapwood of the shoot and that this cross-sectional area was propagated down through all more proximal shoots, branches, and the trunk. Based on empirical investigation, the sapwood area per unit foliage requirement declined with increasing branching order. The length, radius, and amount of foliage on new shoots were in proportions derived from empirical investigations. Segments became shorter as branching order increased. The length, and consequently other dimensions, was determined by the availability of photosynthate. After considering respiration losses, the photosynthate available for growth was considered in one pool and all of it distributed to growth.

The model simulated growth and development of trees over four decades giving effective 3-D images. However, Sievänen et al. (2008) noted a number of discrepancies from expected quantities. Generally, branch diameters and branch lengths were greater than expected, and the number of surviving branches was greater. Branch mortality in the model only occurred close to the base of the live crown, whereas field studies showed it to be distributed over more of the crown length. Increasing the density of trees in the plot decreased tree diameter as expected. An interesting result was that increase in foliage density along shoots caused lower photosynthesis per unit mass. Shoot extension was greater with lower foliage mass which increased both photosynthesis, by decreasing crowding, and production of woody material.

Sievänen et al. (2008) commented that considering the resources available for growth in one pool may not be appropriate. If resources are low then all growing segments grow less so that, for example, a branch producing less than it consumes in respiration decreases the growth of branches that produce a surplus. This does not agree with branch autonomy (Sprugel et al., 1991). Sievänen et al. (2008) noted that the larger dimensions of branches produced by the model than found in measurements may be due to inadequacy in the light model component or application of the pipe model.

Competition for a number of species (*Fagus sylvatica*, Letort et al., 2008; *Pinus tabulaeformis*, Guo et al., 2012) have been studied using the GREENLAB model (Yan et al., 2004) which comprises a formal grammar to describe plant structure. Mathieu et al. (2009) presented a version where increment to the plant body depends upon the ratio of biomass produced to demand from new meristems. The plant grew as a collection of sinks competing for allocation of photosynthate. Net photosynthate production was calculated from radiation interception which depended upon a calibrated radiation use efficiency and a coefficient related to the projected ground surface area of the plant. Biomass was stored in a common pool and distributed among new and existing organs according to calculated sink values. Distribution to the cambium was computed according to the pipe-model theory. A feedback between growth and development was included whereby the number of branches and the composition of growth units depended upon the ratio of the increment pool of biomass/plant demand which is the sum of all sinks in the current growth cycle.

Plants were simulated in a homogenous stand and competition was the result of shading which affects biomass increment and, as plant density increases, a greater priority in allocation was given to height growth. Mathieu et al. (2009) discussed four issues. First, organogenesis may not be strictly controlled by the ratio of available biomass to demand. Second, the inability of the model to reproduce the spatially heterogeneous expression of plasticity, which they comment cannot be neglected in large trees. Third, the hypothesis of a common pool of photosynthate may not be adequate. Fourth, in some instances the supply of photosynthate may be regulated by demand.

### CANOPY DEVELOPMENT, LIGHT INTERCEPTION, AND COMPETITION

Models that simulate penetration of light into canopies based on absorption, reflection, and transmission for individual structural elements of the canopy particularly that of Chelle and Andrieu (1998) have stimulated research into canopy development and competition. When combined with a model for detailed geometry of the foliage canopy estimates can be made of light conditions at the organs of individual plants and their light-dependent growth. In the two cases reviewed here, the investigators reported advances in understanding provided by modeling as well as improvements they consider should be made to models.

From empirical investigations with spring wheat Evers et al. (2006) reported that the probability of tiller appearance decreased earlier in crop development at higher population density. Their simulation study (Evers et al., 2007) was designed to investigate the form of the relationship between R:FR and the relative extension of a tiller bud. Evers et al. (2007) used an architectural model, ADEL-wheat (Fournier et al., 2003) calibrated for spring wheat (Evers et al., 2006, 2007) and the nested radiosity light interception model (NR) of Chelle and Andrieu (1998). ADEL-wheat simulates production and growth from a given initial planting of phytometer units comprising a leaf (blade and sheath) inserted on a node, an internode, and a tiller bud. ADEL-wheat calculates leaf size and shape, basal angle, and curvature, and blade and tiller azimuth angles. Each leaf is defined by a set of polygons with coordinates that establish their position in space. This geometry was interfaced to NR which calculates irradiance on, and energy absorbed by, each simulated plant organ.

Evers et al. (2007) hypothesized that the growth of tiller buds was arrested when R:FR received fell below a threshold value. Tiller bud extension was represented in ADEL-wheat by an exponential growth function

$$L=L_{0}e^{F.RFR_{p}.t},$$

where *L* is the bud length, *L*_0_ is the initial bud length, RER_p_ is the potential relative extension rate (^o^Cd)^-^^1^, *t* is the thermal time (^o^Cd) since the initiation of the bud. *F* was a function of R:FR so that *F* decreased as R:FR decreased and three forms were examined, a threshold value and curvilinear or linear decrease.

Simulations were conducted for a range of initial planting for which the time course of numbers of tillers.plant^-^^1^ had been measured in field experiments. Evers et al. (2007) noted that a threshold function with an R:FR value of 0.8–0.9 was required to simulated both a comparable tillering rate to field data and a final tiller number to that found experimentally. This was considerably higher than found in measurements and they suggest this may be due to use of a higher above canopy R:FR, 1.2, than is actually found under natural conditions.

Dauzat et al. (2008) combined the modeling strategy of using an architectural and a light penetration model with analysis of canopy structure using the 3-D Fastrack digitizer (Polhemus Inc., Sinoquet and Rivet, 1997). They studied growth of cotton at three densities, 1, 2, and 4 plants m^-^^2^. They anticipated that greater morphogenetic plasticity would occur at higher planting densities and investigated the effects of these changes on crop efficiency in intercepting light over a growing season. Measurements were made of crop cover, time courses of plant height growth, and leaf area.

They observed that morphogenetic plasticity varied with plant density and stage of development of the stand with a transition between increasing internode lengths in the lower part of the stem and decreasing internode lengths in the upper part. Dauzat et al. (2008) suggested that while the pattern of increasing internode lengths early in development was consistent with decreasing R:FR this was counteracted by plant carbon limitations during the latter phase. They noted that internode length and leaf area increment decreased simultaneously. The transition between the two phases occurred when average leaf irradiance decreased below 60% of incident PAR.

## CONCLUSION

Although it seems intuitively obvious that plant architecture should affect competition for light between plants we have little knowledge about the effects that different architectural features may have on the competition process. Not surprisingly, pioneering studies that demonstrated such effects (Ellison and Rabinowitz, 1989; Geber, 1989) used plants with large contrasts. Niklas (1997) description of how multiple plant forms may have arisen suggests that obtaining an understanding of competition effects may be a considerable challenge. Nevertheless, explaining competition effects is one of the important challenges faced by FSPMs because of the range of information that must be integrated into a model.

Much research into competition has been conducted with a standpoint of population biology and such work enables us to define some effects of competition on stands and individual plants. However, it has not provided an analytical framework for analysis of the effects of architecture on the process. Studies of plant functioning in stands undergoing competition have shown interesting responses to changes in the environment as a stand develops. However, the assessment of effects on competition in such work has typically been limited to measurements that only give a partial representation of the competition process and interactions between different aspects of physiology and morphology have largely been ignored.

Section “Development of Theory for the Effects of Plant Architecture on Competition,” of this review outlines a basic structure of a theory for analyzing the influences that architecture may have on competition and how such a theory may be assessed. The research reviewed here was conducted on multiple species growing under different conditions. The proposed theory is a synthesis from this wide ranging work. Postulates for two groups of ideas are central. Group I—that competition for light takes place at the level of foliage and foliated axes—defines competition as a local event within the canopy. To a considerable extent, it tallies with theories of plant growth based on modular development of plants and branch and foliage reiteration. Use of this postulate in models for competition would reorient how such models are constructed.

Group II defines the outcome of competition as determined by the interaction between the exertion of dominance, particularly through resource acquisition plasticity, and reaction to shading through changing form and/or physiological characteristics. This suggests it is essential to study *interactions* between these processes rather than just one or another. The pioneering work of Casal et al. (1986) did this, at least to the extent of showing the importance of a certain level of PAR being necessary for a response to changes in R:FR. It was unfortunate that this duality in approach was not continued as it led to larger claims being made for the importance of morphogenetic plasticity than are warranted. The field study of Dauzat et al. (2008) showing a change over time in the control of internode length, from R:FR to carbohydrate limitation reinforces the need for this approach.

Work with tree species illustrates that considerable development of the Group 4 postulates on integration of plant growth is essential. Although it seems reasonable that competition for light should be accounted at the level of foliage and the foliage-bearing structure, understanding and representing how such units are sustained requires considerable work. The idea of a whole-plant carbon pool and the pipe-model theory for addition of wood need to be replaced and this suggests that further research is required into the interactions between carbohydrate metabolism and the structures produced for water conduction.

Section “Development of Theory for the Effects of Plant Architecture on Competition” illustrates the value of models, when combined with field investigations. Laboratory-based investigations present us with possibilities that certain processes may be important—but the experimental conditions under which they are established may not reflect those found in developing stands. Stand conditions do need to be documented carefully.

## Conflict of Interest Statement

The author declares that the research was conducted in the absence of any commercial or financial relationships that could be construed as a potential conflict of interest.
